# A Dual‐Modal Wearable PPG Smartwatch with AI‐Enhanced Correction for High‐Accuracy and Continuous AF Burden Assessment

**DOI:** 10.1002/advs.76627

**Published:** 2026-07-14

**Authors:** Song Zuo, Jinglei Wang, Xin Wang, Han Feng, Yize Zhao, Fadong Li, Shaobin Wei, Le Zhou, Zixu Zhao, Songjie Chen, Xiangyi Kong, Jue Wang, Liu He, Shijun Xia, Xin Li, Junmeng Zhang, Xiaoxia Liu, Xueyuan Guo, Ning Zhou, Songnan Li, Chenxi Jiang, Ribo Tang, Caihua Sang, Deyong Long, Xin Du, Jianzeng Dong, Wenwu Zhu, Changsheng Ma

**Affiliations:** ^1^ Department of Cardiology, Beijing Anzhen Hospital, Capital Medical University National Clinical Research Center for Cardiovascular Diseases Beijing China; ^2^ Xinjikang Technology Co., Ltd Chengdu China; ^3^ Department of Computer Science and Technology, National Research Center for Information, Science and Technology Tsinghua University Beijing China; ^4^ Cardiology Department Tulane University School of Medicine New Orleans Louisiana USA

**Keywords:** AF burden, AI‐correction, convolutional neural networks, long short‐term memory, photoplethysmography

## Abstract

Atrial fibrillation (AF) increases the risk of stroke and heart failure, yet accurate quantification of AF burden in daily life remains difficult. Although smartwatch photoplethysmography (PPG) supports continuous monitoring, complex rhythms and signal noise can impair burden estimation. We developed an AI‐enhanced dual‐modal framework that combines continuous watch‐based PPG (W‐PPG) with intermittent single‐lead watch‐based ECG (W‐ECG). A hybrid convolutional neural network–long short‐term memory model uses high‐fidelity W‐ECG segments as dynamic anchors to correct long‐term W‐PPG classifications. In this prospective validation study, 1,054 patients with AF undergoing catheter ablation (mean age, 62.1 years) were evaluated against patch‐based ECG as the reference standard. After ECG‐based correction, the system achieved 98.60% sensitivity and 99.27% specificity. The mean absolute percentage error of AF burden decreased by 23.4%, from 1.11% to 0.85%, while the Pearson correlation remained 0.9988. This dual‐modal approach offers a scalable and clinically practical solution for long‐term AF monitoring, improving burden estimation beyond PPG‐only devices without requiring continuous multi‐lead ECG. It may support personalized AF management and large‐scale cardiovascular screening in real‐world settings. (NCT06552468)

## Abstract 2

This figure illustrates the hardware, data processing workflow, and diagnostic performance of the dual‐modal wearable system integrating smartwatch PPG/ECG and patch‐based ECG with AI correction. (A) Exploded view of the patch components, including upper and lower shells, power button, battery, main PCB, and light PCB. (B) Exploded view of the smartwatch components, including screen, main PCB, sensor switch, battery, housing, sensor PCB, back cover, and electrodes. (C) Workflow showing simultaneous W‐PPG and W‐ECG signal acquisition. W‐ECG was used to correct W‐PPG via feature extraction and deep learning–based AF classification, and patch‐based ECG (P‐ECG) was used as the gold standard for evaluation. (D) Radar plot comparing model performance before (blue) and after (orange) AI correction. Metrics include AUC, SE, SP, PPV, NPV, ACC, and F1 score, demonstrating substantial improvement after correction. Abbreviations: AF: Atrial Fibrillation; W‐ECG: Watch‐based Electrocardiogram; W‐PPG: Watch‐based Photoplethysmography; P‐ECG: Patch‐based Electrocardiogram; AI: Artificial Intelligence; SE: Sensitivity; SP: Specificity; PPV: Positive Predictive Value; NPV: Negative Predictive Value; ACC: Accuracy.

## 1 Introduction

Atrial fibrillation (AF) is the most common sustained cardiac arrhythmia encountered in clinical practice. Pathophysiologically, it is characterized by rapid and highly disorganized atrial electrical activity, which results in the loss of effective atrial mechanical contraction and gives rise to an absolutely irregular ventricular rhythm [1, 2, 3]. With global population aging, the prevalence of AF has risen dramatically and now represents a major public health burden [4, 5]. The latest Global Burden of Disease (GBD) study showed that the number of individuals with AF and atrial flutter increased from 22.2 million in 1990 to 59.7 million in 2021, representing an increase of more than 160% [4]. Projections further suggest that the prevalence of AF will continue to increase and may even double by 2050, thereby posing a profound challenge to global healthcare systems in this century [6].

The clinical burden of AF extends far beyond the arrhythmia itself and is largely driven by its association with a series of severe complications [7, 8]. Patients with AF have an approximately fivefold higher risk of ischemic stroke than those without AF, and AF‐related strokes are generally associated with greater disability and mortality. Previous studies have shown that approximately 20%–30% of ischemic strokes are directly related to either subclinical or previously diagnosed AF [9, 10]. In addition, AF and heart failure exhibit a complex and tightly intertwined bidirectional relationship. AF increases the risk of heart failure by approximately threefold and is associated with a marked increase in all‐cause mortality [11]. In recent years, the adverse impact of AF on neurocognitive function has also attracted increasing attention. Evidence indicates that AF is associated with a 39% increase in the risk of cognitive impairment and a 1.4‐ to 2.2‐fold higher risk of dementia, including Alzheimer's disease. This association remains significant even after adjustment for clinical stroke, suggesting that microembolism and cerebral hypoperfusion may play important pathogenic roles [12, 13].

Beyond its substantial impact on disease risk, AF also imposes a considerable economic burden. Studies have shown that the annual per‐patient healthcare expenditure in individuals with AF is approximately US$6000–12 000 higher than that in those without AF, with more than 70% of the total cost attributable to hospitalization and the management of related complications [14, 15]. However, because AF is often highly occult, particularly in the setting of asymptomatic AF, many patients remain undiagnosed until severe adverse events such as stroke occur [16, 17, 18]. Therefore, the development of highly accurate early screening technologies and continuous dynamic monitoring strategies is of critical importance not only for optimizing individualized treatment and preventing adverse cardio‐cerebrovascular events, but also for alleviating the growing global healthcare and economic burden.

At present, the diagnosis and assessment of AF still rely predominantly on short‐term event‐detection tools, such as 12‐lead electrocardiography (ECG) and 24 h Holter monitoring [19, 20, 21]. Although Holter monitoring can capture AF episodes occurring over a short observation window, its limited monitoring duration makes it insufficient for reflecting round‐the‐clock and long‐term variations in rhythm burden. Implantable cardiac electronic devices, such as implantable cardioverter‐defibrillators or continuous rhythm monitors, can provide longer‐term continuous recordings; however, their invasiveness, high cost, and procedural complexity limit their broad applicability in large‐scale population screening [22, 23, 24]. More importantly, AF is not merely a binary condition defined by the presence or absence of arrhythmia, but rather a dynamic disease with continuous‐spectrum characteristics [23, 25]. AF burden, defined as the proportion of cumulative AF duration over the total monitoring time, is closely associated with long‐term adverse outcomes, including stroke, heart failure, and mortality [25, 26]. Nevertheless, current clinical practice and most existing monitoring technologies remain largely focused on event identification, whereas precise quantitative assessment of AF burden remains insufficient.

With the rapid development of wearable devices and artificial intelligence (AI), AF screening based on portable, low‐cost, and continuously operating monitoring platforms has emerged as an important research direction in this field [27, 28, 29]. Photoplethysmography (PPG) is a noninvasive optical biosensing technique that indirectly reflects cardiovascular dynamics by detecting changes in light reflection caused by cyclic fluctuations in microvascular blood volume [30, 31, 32, 33]. Because PPG can be readily integrated into wearable terminals such as smartwatches and enables continuous monitoring with minimal user burden, it has shown considerable promise for AF screening and long‐term management [34, 35, 36]. Pereira et al. reported that PPG‐based monitoring strategies combined with signal processing and machine learning/deep learning algorithms have already demonstrated favorable performance in AF detection and hold promise for large‐scale public health screening [37].

More recently, smartwatch‐based PPG algorithms have also been explored for quantitative AF burden assessment. Wang et al. developed and validated a PPG‐based machine learning model for AF burden estimation using smartwatch monitoring and demonstrated good concordance with Holter‐derived AF burden metrics, supporting the potential of wearable PPG for noninvasive and convenient tracking of AF progression [38]. In addition, the DoubleCheck‐AF study evaluated a wrist‐worn device integrating continuous PPG‐based AF screening with instant ECG rhythm confirmation and showed that the addition of ECG could help maintain high specificity, particularly in patients with frequent premature contractions [39]. These recent findings further support the clinical value of wearable PPG monitoring while highlighting the importance of ECG‐assisted confirmation or correction to improve diagnostic reliability in real‐world settings.

Methodologically, PPG‐based AF detection mainly relies on the capture of pulse interval variability and other time‐domain and frequency‐domain features, which are subsequently combined with conventional statistical methods or machine learning models to discriminate AF from sinus rhythm [37, 40]. However, PPG signals are highly susceptible to motion artifacts, ambient light interference, and nontarget physiological noise, thereby limiting detection accuracy and robustness [30, 41, 42]. To improve recognition performance under complex real‐world conditions, deep learning approaches have been widely introduced into PPG signal analysis [43, 44, 45]. Aldughayfiq et al. developed a hybrid architecture integrating convolutional neural networks (CNNs) and long short‐term memory (LSTM) networks to classify AF by fusing ECG and PPG time‐series data, achieving approximately 95% accuracy, 88% precision, and 85% sensitivity in the test set [46]. These findings indicate that CNNs can automatically extract rich local features from raw time‐series signals, including waveform morphology and instantaneous amplitude variation, whereas LSTMs are well suited for modeling long‐range temporal dependencies, such as the persistent evolution of irregular rhythm patterns. Together, these complementary capabilities substantially enhance the robustness of AF recognition.

Similarly, Aschbacher et al. compared multiple analytical strategies for AF detection using PPG‐derived data and showed that, whereas logistic regression analysis based on heart rate variability achieved an area under the receiver operating characteristic curve (AUC) of 0.717 (sensitivity, 0.741; specificity, 0.584), the LSTM model trained on heart rate data improved the AUC to 0.954 (sensitivity, 0.810; specificity, 0.921). Notably, the deep neural network trained directly on raw PPG waveforms yielded the best performance, with an AUC of 0.983, sensitivity of 0.985, and specificity of 0.880 [47]. This further highlights that deep neural networks can not only automatically learn key discriminative patterns embedded in PPG signals, but also suppress noise interference to a certain extent, making them particularly suitable for continuous monitoring in dynamic environments.

The application of PPG in practical wearable devices has also gradually entered the stage of clinical validation. Mobile health applications such as FibriCheck, through joint analysis of PPG and single‐lead ECG signals, achieved approximately 96% sensitivity and 97% specificity for AF screening in primary care populations [48]. In addition, several recent studies have reported that CNN‐based PPG models can achieve real‐time AF detection with accuracy and sensitivity exceeding 98%, further underscoring the potential of integrating deep learning with PPG for wearable AF monitoring [49].

Despite these advances, current studies and applications still face several substantive challenges. First, PPG signals are highly sensitive to motion artifacts and ambient light interference, resulting in insufficient detection stability. Second, the performance of existing algorithms often deteriorates substantially in high‐noise scenarios, such as vigorous movement or complex rhythm disturbances. Finally, most currently available PPG‐only strategies remain primarily focused on AF event detection, whereas investigations into the continuous and precise quantification of AF burden remain relatively limited. Collectively, these limitations have markedly constrained the clinical translation of PPG technology for large‐scale screening and long‐term continuous dynamic monitoring.

To address the limitations of existing AF monitoring strategies, we developed an AI‐enhanced multimodal smartwatch system that combines continuous PPG monitoring with ECG‐assisted correction to enable more continuous and accurate quantification of AF burden. The system employs a deep learning framework integrating convolutional neural network (CNN) and long short‐term memory (LSTM) architectures for precise interpretation and dynamic correction of multimodal signals. In this framework, PPG serves as the primary modality for long‐term, continuous rhythm surveillance, whereas ECG provides high‐fidelity electrophysiological reference information for calibration. Specifically, the CNN module automatically extracts high‐level local features from the signals, including waveform morphology and beat‐to‐beat interval fluctuations, thereby reducing reliance on handcrafted feature engineering, and improving the accuracy of AF recognition and assessment. The LSTM module further captures long‐range temporal dependencies within the time‐series data, making it particularly suitable for identifying persistent irregular rhythm patterns and their temporal evolution, and thereby enhancing the fine‐grained evaluation of AF burden.

To further improve monitoring accuracy and robustness, the smartwatch is additionally equipped with a single‐lead ECG module, which acquires ECG signals through three complementary modes: scheduled ECG triggering, event‐triggered ECG acquisition following positive screening results, and user‐initiated ECG recording. These ECG recordings are used to calibrate and correct PPG‐derived AF burden estimates under real‐world conditions. Because PPG signals are inherently susceptible to motion artifacts, premature beats, and other nonspecific interferences, the inclusion of ECG enables verification and refinement of PPG‐based results, thereby improving both reliability and quantitative precision. Through the joint analysis of continuously acquired PPG signals and intermittently recorded ECG signals, this multimodal system achieves more continuous, accurate, and robust AF burden monitoring than single‐modality approaches.

Overall, the system developed in this study consists of two independent but complementary modules: a smartwatch module for signal acquisition and a cloud‐based algorithmic module for computational analysis. Together, these modules enable precise, reliable, and continuous dynamic assessment of AF burden and are well suited for long‐term monitoring in real‐world settings. By combining the continuity and scalability of PPG with the diagnostic fidelity of ECG, together with CNN‐ and LSTM‐based signal correction, this approach has the potential to overcome the limitations of traditional AF monitoring strategies in timeliness, accessibility, and quantitative capability. It may therefore provide an efficient, user‐friendly, and clinically translatable solution for future AF screening, burden assessment, risk stratification, and long‐term health management.

## 2 Results and Discussion

### 2.1 Design of the Dual‐Modal Wearable Monitoring System

Clinicians typically assess irregular cardiac electrical activity using standard ECG, while simultaneously analyzing pulse rate variability and rhythm irregularity from PPG signals. This combined approach enables a quantitative evaluation of AF burden, defined as the percentage of time spent in AF over the total analyzable recording duration. Inspired by the synergistic monitoring of multimodal physiological signals, we developed a dual‐modal wearable monitoring system for continuous and precise AF surveillance. This system, provided by Chengdu Xinjikang Technology Co., Ltd., consists of two core components: a wrist‐based ECG acquisition module (W‐ECG) and a wrist‐based PPG acquisition module (W‐PPG) (Figure 1B). By integrating PPG and single‐lead ECG signals within a smartwatch platform, the system enables uninterrupted PPG monitoring together with user‐initiated ECG recording on demand, thereby overcoming the short‐term limitations of conventional Holter monitoring and reducing the cost burden associated with invasive devices.

**FIGURE 1:**
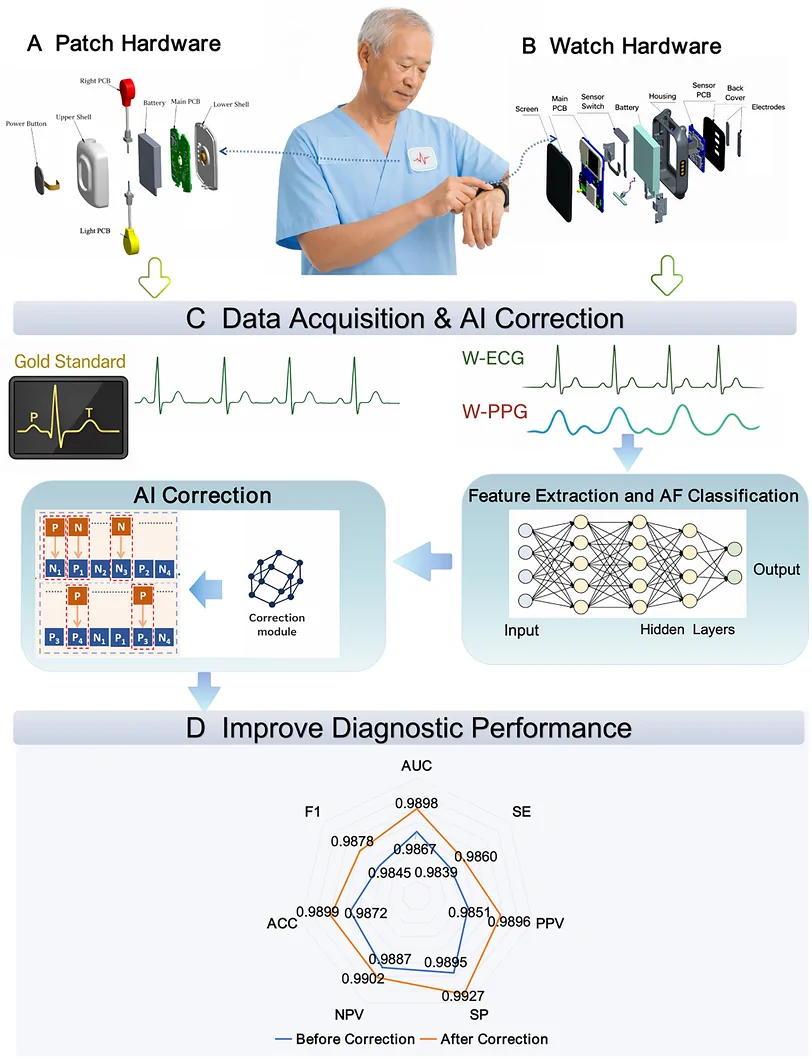
AI‐Enhanced Dual‐Modal Smartwatch and Patch System for Accurate Atrial Fibrillation Burden Assessment. This figure illustrates the hardware, data processing workflow, and diagnostic performance of the dual‐modal wearable system integrating smartwatch PPG/ECG and patch‐based ECG with AI correction. (A) Exploded view of the patch components, including upper and lower shells, power button, battery, main PCB, and light PCB. (B) Exploded view of the smartwatch components, including screen, main PCB, sensor switch, battery, housing, sensor PCB, back cover, and electrodes. (C) Workflow showing simultaneous W‐PPG and W‐ECG signal acquisition. W‐ECG was used to correct W‐PPG via feature extraction and deep learning–based AF classification, and patch‐based ECG (P‐ECG) was used as the gold standard for evaluation. (D) Radar plot comparing model performance before (blue) and after (orange) AI correction. Metrics include AUC, SE, SP, PPV, NPV, ACC, and F1 score, demonstrating substantial improvement after correction. Abbreviations: AF: Atrial Fibrillation; W‐ECG: Watch‐based Electrocardiogram; W‐PPG: Watch‐based Photoplethysmography; P‐ECG: Patch‐based Electrocardiogram; AI: Artificial Intelligence; SE: Sensitivity; SP: Specificity; PPV: Positive Predictive Value; NPV: Negative Predictive Value; ACC: Accuracy.

#### 2.1.1 Watch‐Based PPG Module (W‐PPG)

The W‐PPG module was integrated into a smartwatch and constructed around an optical sensing system optimized for stable long‐term acquisition. To improve light penetration and enhance signal fidelity, the optical probe was tailored to the optical properties of human skin and operated at a sampling frequency of 25.6 Hz. The probe adopted a reflective optical architecture and incorporated a multi‐wavelength LED source, including a 525 nm green LED, together with multiple large‐area photodiode receivers, thereby enabling sensitive detection of pulsatile blood volume changes.

To support prolonged wearable use, the smartwatch back cover was fabricated from biocompatible materials to ensure skin safety and wearing comfort, while the silicone strap provided flexibility, breathability, and skin compatibility. During operation, strap tightness could be adjusted to maintain stable skin contact and improve signal‐to‐noise ratio, while avoiding excessive compression of superficial capillaries that might otherwise distort the PPG waveform. The module was built on a low‐power MAX86176 pulse‐sensing platform embedded in an Android‐based smartwatch. Under continuous monitoring conditions, the Bluetooth unit and main controller consumed an average current of ≈7 mA, whereas the total system current was ≈10 mA, with W‐PPG accounting for the major share of power consumption. This power‐management strategy supported continuous operation for more than 24 h, together with rapid recharging within ≈1 h.

#### 2.1.2 Watch‐Based ECG Module (W‐ECG)

The W‐ECG module incorporated two dry stainless‐steel electrodes: one located on the back of the watch case, remaining in continuous contact with the wearer's wrist, and the other positioned on the front/upper side of the case, contacted by a finger of the opposite hand to complete the measurement circuit. A separate capacitive touch switch, activated by the opposite‐hand thumb, was used to initiate and terminate ECG recording (Figure 2C). ECG signals were acquired at 512 Hz. This design generated a potential difference between the left and right upper limbs and was therefore functionally equivalent to a standard lead I ECG configuration.

**FIGURE 2:**
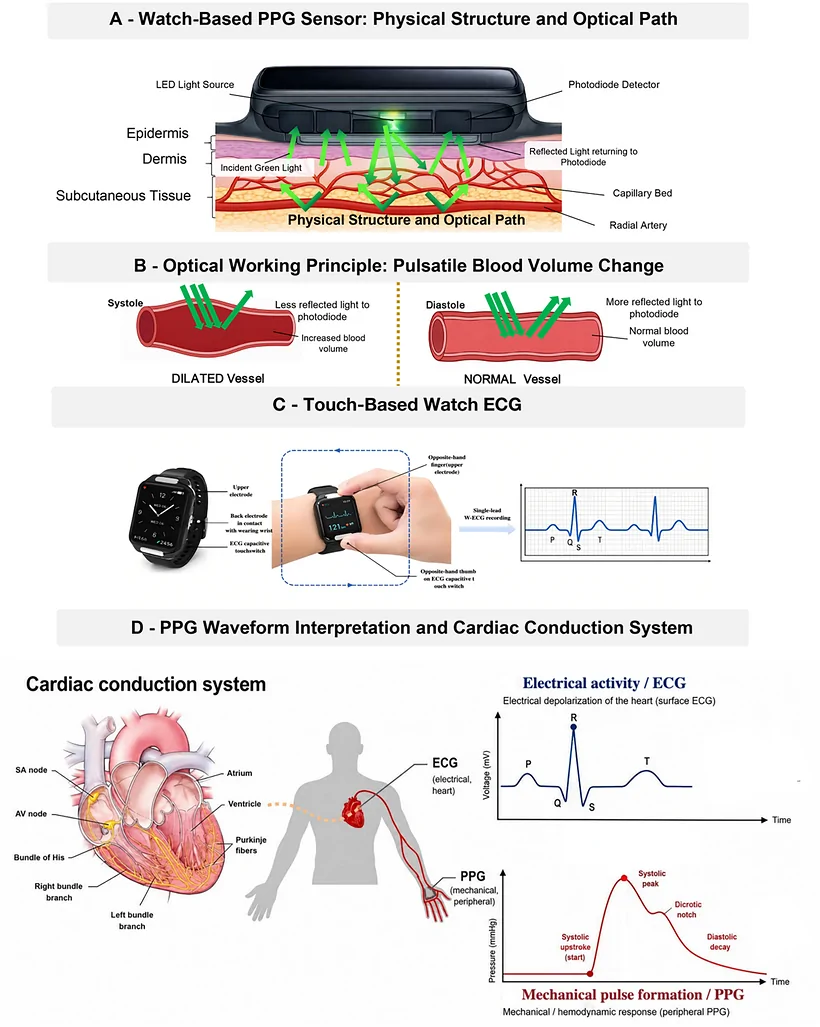
Smartwatch‐Based PPG Sensor and Touch‐Based ECG: Structural and Functional Overview. This figure illustrates the physical structure, optical working principle, ECG acquisition, and interpretation of systolic and diastolic components in the smartwatch‐based cardiovascular monitoring system. (A) Watch‐Based PPG Sensor: Physical Structure and Optical Path. Exploded view of the smartwatch PPG sensor, showing the LED light source, photodiode detector, and light propagation through the epidermis, dermis, and subcutaneous tissue to detect radial artery pulsation. (B) Optical Working Principle: Pulsatile Blood Volume Change. Schematic illustrating how changes in blood volume during systole and diastole affect reflected light intensity, forming the basis for PPG signal acquisition. (C) Touch‐Based Watch ECG. Illustration of single‐lead ECG acquisition via two dry electrodes (an upper/front electrode contacted by the opposite‐hand finger, and a back electrode in contact with the wearing wrist), activated by an ECG capacitive touch switch, and the resulting single‐lead W‐ECG waveform (P, Q, R, S, and T). (D) PPG Waveform Interpretation and Cardiac Conduction System. Conceptual diagram illustrating the physiological interpretation of the PPG waveform, showing the systolic and diastolic components in the context of cardiac electrical conduction and pulse generation. Abbreviations: PPG: Photoplethysmography; ECG: Electrocardiogram; SA node: Sinoatrial Node; AV node: Atrioventricular Node.

To improve practicality in everyday use, the smartwatch incorporated a rapid‐start mechanism that allowed W‐ECG recording to be initiated by a simple touch, enabling convenient operation and efficient signal acquisition in daily‐life scenarios. Unlike most existing smartwatches that restrict each W‐ECG recording to a fixed 30 s duration, the present device supported extended recordings beyond 30 s according to user habits and actual monitoring needs, thereby allowing the capture of richer electrophysiological information.

#### 2.1.3 Patch‐Based ECG Module (P‐ECG)

The P‐ECG module employed a flexible patch‐based single‐lead device designed for extended wear and continuous ECG acquisition in clinical settings. The device incorporated standard electrode interfaces and an ergonomically optimized structure, including refined electrode layout and fitting curvature, to improve wearing stability on the chest surface (Figure 1A). By integrating highly sensitive flexible electrodes with an ultra‐low‐power analog front‐end circuit, the system enabled stable ECG acquisition at a sampling frequency of 250 Hz and ensured reliable capture of irregular cardiac rhythms.

To reduce baseline drift and external interference, standardized skin preparation was performed before use, including hair removal, and degreasing with 75% alcohol. The long‐endurance performance of this module relied on an MCU‐based architecture combined with an ultra‐low‐power analog front‐end chip, with a typical average current of ≈1.5 mA under a 3.3 V supply. This configuration supported continuous wear for up to 7 days and was therefore suitable for prolonged clinical monitoring. Functionally, this acquisition mode was comparable to conventional Holter recording, and the collected data were used as the gold standard throughout the study.

#### 2.1.4 Communication and Time‐Synchronization Module

The communication and synchronization module comprised a lithium battery pack and a Bluetooth Low Energy (BLE) interface, enabling precise temporal synchronization across devices. The smartwatch and patch were paired in real time via the BLE protocol, with timestamp alignment applied to ensure accurate correspondence between signals during subsequent analyses, thereby minimizing the influence of temporal drift on RR interval or pulse‐peak interval assessment. In addition, this module supported low‐power wireless signal transmission, preserving the overall energy‐efficient characteristics of the system.

#### 2.1.5 System‐Level Collaborative Acquisition Strategy and Monitoring Principle

Based on the above hardware design, we established a smartwatch‐centered dual‐modal monitoring strategy that enabled coordinated acquisition of W‐PPG and W‐ECG, thereby providing complementary physiological information for continuous AF monitoring and burden assessment. Specifically, W‐PPG served as the primary modality for long‐term, low‐burden pulse‐wave acquisition and captured pulse rate variation and rhythm irregularity, whereas W‐ECG provided short‐duration yet high‐specificity electrophysiological information and functioned as a real‐time anchor for signal correction. By combining these two modalities, AF‐related features could be identified jointly from both hemodynamic and electrophysiological dimensions, thereby improving the accuracy of AF detection and burden estimation.

At the signal‐analysis level, AF is typically manifested in PPG as markedly irregular pulse intervals and loss of rhythmic consistency, whereas in ECG it is characterized primarily by the absence of P waves and absolute irregularity of RR intervals. On this basis, the system adopted a collaborative strategy of continuous W‐PPG monitoring with key W‐ECG correction, in which W‐PPG served as the main source for long‐term dynamic surveillance and W‐ECG acted as a correction reference to reduce false‐positive and false‐negative classifications caused by motion artifacts, pulse deficits, premature beats, and other sources of interference. Through joint modeling of these two signal types using deep learning algorithms, the system enabled more accurate identification of AF episodes and more reliable estimation of AF burden.

In addition, the P‐ECG module provided highly reliable single‐lead ECG recordings, and the acquired data were used as the gold standard to validate the accuracy of the smartwatch‐based dual‐modal monitoring results and performance evaluation. Collectively, this system established a smartwatch‐centered dual‐modal collaborative monitoring framework, and thus provided a technical foundation for long‐term, noninvasive, and precise AF monitoring (Figure 1C).

### 2.2 Characterization of W‐PPG and W‐ECG Detection Principles

#### 2.2.1 Detection Principle of W‐PPG

PPG is a noninvasive optical sensing technique that indirectly reflects cardiac activity by detecting cyclic changes in microvascular blood volume over the cardiac cycle. In this study, the W‐PPG module was integrated into the underside of the smartwatch and implemented in a reflective optical configuration. A green LED with a primary wavelength of 525 nm was used as the light source to illuminate the skin tissue. Part of the emitted light was absorbed by hemoglobin, whereas the remaining light was scattered and reflected by the tissue before being captured by multiple large‐area photodiode receivers. The optical design, including the wavelength selection and light intensity setting, was optimized according to the optical properties of human skin, such as absorption and scattering, to ensure effective penetration into the superficial tissue layer and maximize signal contrast (Figure 2A).

During systole, increased arterial blood volume enhances light absorption and reduces reflected intensity. During diastole, the opposite occurs, producing cyclic changes that photodiodes convert into the PPG waveform (Figure 2B). These cyclic changes in light intensity are converted by the photodiode receivers into electrical signals, thereby generating the PPG waveform. Both the frequency and morphology of the waveform directly correspond to rhythm dynamics and peripheral pulse behavior (Figure 2D).

Several characteristic morphological landmarks can be identified in the PPG waveform. The systolic peak represents the highest point of the waveform and corresponds to maximal peripheral arterial expansion. The dicrotic notch appears as a downward inflection on the descending branch and is associated with aortic valve closure, often being more pronounced in young individuals with preserved vascular elasticity. The diastolic peak is a secondary peak following the dicrotic notch and is mainly attributed to reflected waves from the peripheral vasculature.

Under sinus rhythm, the pulse‐to‐pulse interval sequence derived from continuous hardware acquisition is highly consistent with the cardiac rhythm, showing low beat‐to‐beat variability and high waveform reproducibility. The raw optical signal exhibits stable periodic oscillations with relatively uniform amplitude. By contrast, under AF, the rapid and disorganized atrial activity gives rise to irregular ventricular response, which is reflected in the PPG signal as pronounced pulse interval irregularity, loss of rhythmic consistency, and the absence of a fixed temporal pattern. In addition, waveform amplitude fluctuates markedly, and the height of the main systolic peaks becomes unstable, reflecting disturbed peripheral hemodynamics. In some cycles, missing peaks or fused peaks may occur, corresponding to rapid ventricular response or premature beats. These signal‐level manifestations form the physiological basis for AF detection using PPG.

To ensure signal quality at the hardware level, optical probes were subjected to factory SNR screening under standardized testing conditions, and only units with SNR > 10 dB were selected for deployment. Because PPG signals are inherently susceptible to multiple sources of interference, the robustness of the W‐PPG module was further characterized experimentally. Motion artifacts, such as those caused by arm movement or muscle contraction, may induce baseline drift and spike‐like noise. To mitigate these effects, the module adopted a silicone strap‐based fixation design to reduce relative displacement between the sensor and skin. In addition, factory screening and experimental validation confirmed that the optical probe maintained stable SNR under standardized illumination and skin‐mimicking conditions, with a mechanical response time of <50 ms, thereby supporting the capture of transient rhythm changes. The system also exhibited stable output across a humidity range of 20%–100% and a temperature range of 20  C–40 °C. Moreover, no obvious deterioration in signal quality was observed after 3 months of storage. Together, these results demonstrate the reliable acquisition capability of the W‐PPG system across different operating conditions and support its suitability for long‐term wearable monitoring.

#### 2.2.2 Detection Principle of W‐ECG

W‐ECG is a bioelectric sensing modality that directly reflects cardiac electrophysiological activity by detecting voltage changes generated during myocardial depolarization and repolarization. In this system, the W‐ECG module employed dry stainless‐steel electrodes to acquire weak cardiac electrical signals through a single‐lead circuit. The acquired signal was processed by a high‐gain, low‐noise analog front‐end circuit, while a driven‐right‐leg circuit was used to suppress common‐mode interference and improve signal clarity. ECG signals were sampled at 512 Hz, meeting the requirements for clinical rhythm monitoring.

The ECG waveform contains several canonical features (Figure 2C). The P wave reflects atrial depolarization and typically exhibits an amplitude of 0.05–0.25 mV. The QRS complex reflects ventricular depolarization, with an amplitude of 0.5–2 mV, and is the most prominent component of the ECG signal, representing ventricular electrical activation associated with contraction. The T wave reflects ventricular repolarization and typically ranges from 0.1 to 0.5 mV.

Under sinus rhythm, the ECG signal shows regular RR intervals, clear waveform morphology, low variability, and stable baseline characteristics. In contrast, under AF, the ECG signal is characterized by the disappearance of distinct P waves or the presence of fibrillatory waves, together with absolute irregularity of RR intervals and the absence of a fixed repetitive pattern. These features can be captured directly and accurately by the high‐sampling‐rate recording hardware. Meanwhile, the baseline fluctuation remains relatively limited and the QRS morphology is generally preserved, unless concomitant conduction abnormalities are present, allowing stable representation of ventricular electrical activity. These electrophysiological characteristics provide the direct basis for AF detection using ECG.

The hardware complies with YY 9706.247‐2021 (equivalent to IEC 60601‐2‐47) for ambulatory ECG systems, achieving >95% QRS detection sensitivity, <30 µV p‐p noise levels, and ±4 ms RR interval accuracy. To further evaluate the reliability of ECG‐based rhythm monitoring, standardized verification procedures were performed, confirming that the device met general requirements for acquisition accuracy, noise suppression, safety, and effectiveness in clinical settings.

The ECG hardware performance was characterized in several aspects. First, for noise suppression, the module incorporated built‐in 50/60 Hz notch filtering and an amplifier with a common‐mode rejection ratio >80 dB, enabling effective suppression of mains interference and other external noise sources. Electromyographic interference was further controlled through hardware thresholding, ensuring clear and stable ECG acquisition. The measured noise level remained below 30 µV p‐p, supporting high‐quality signal collection. Second, for long‐term stability, simulated continuous‐use testing showed that QRS amplitude attenuation was <3%, indicating robust performance over prolonged operation. Stable hardware performance was maintained across varying humidity and temperature gradients, in compliance with the service‐life requirements specified in YY 9706.247‐2021 (equivalent to IEC 60601‐2‐47). Third, for population adaptability, testing across individuals of different sex, body weight, and skin type showed that QRS amplitude was not significantly associated with sex or body weight. Although R‐wave amplitude was slightly reduced in obese participants, it remained sufficient for diagnostic use. In addition, signal quality did not exhibit significant degradation under sweating conditions, and the waterproof performance satisfied the corresponding liquid‐protection requirements, supporting stable operation in diverse real‐world scenarios

In the present analysis, 3280 W‐ECG recordings were included, with 740 participants contributing at least one recording. The mean number of W‐ECG recordings was 3.11 ± 3.37 per participant, with a median of 2 and a range of 0–15 recordings.

#### 2.2.3 Synergistic Roles of W‐PPG and W‐ECG

The principal advantage of the dual‐modal system arises from the complementary strengths of W‐PPG and W‐ECG. While W‐PPG enables continuous optical monitoring suitable for prolonged rhythm surveillance, W‐ECG supplies high‐fidelity electrophysiological data that serve as anchors for verification and correction of PPG‐derived measurements. The integrated platform complies with GB 9706.225‐2021 (equivalent to IEC 60601‐2‐25), ensuring consistent stability and accuracy during real‐world operation.

On this basis, the dual‐modal system enables effective AF monitoring by combining continuous hemodynamic sensing with high‐specificity electrophysiological anchoring. This design not only improves detection accuracy and robustness through multimodal integration, but also overcomes, to a certain extent, the limitations of conventional short‐term monitoring approaches. In clinical practice, the system offers a more comfortable and practical solution for prolonged rhythm surveillance, while providing clinicians with more reliable data for early AF screening and precise management.

### 2.3 Mechanistic Basis of AF and Characteristic Changes in PPG Signals

PPG reflects the optical modulation of peripheral microvascular blood volume over the cardiac cycle, with each pulse peak corresponding to the mechanical pulsation transmitted to the periphery. Under sinus rhythm, cardiac electrical activity is governed by the sinoatrial node in a regular manner, resulting in stable systemic hemodynamics. Accordingly, the PPG waveform exhibits highly periodic oscillation with a typical biphasic profile, including a primary systolic wave and a reflected diastolic component. The peak‐to‐peak intervals remain highly consistent and beat‐to‐beat amplitude variation is minimal, collectively indicating stable blood flow and rhythm dynamics (Figure 3).

**FIGURE 3:**
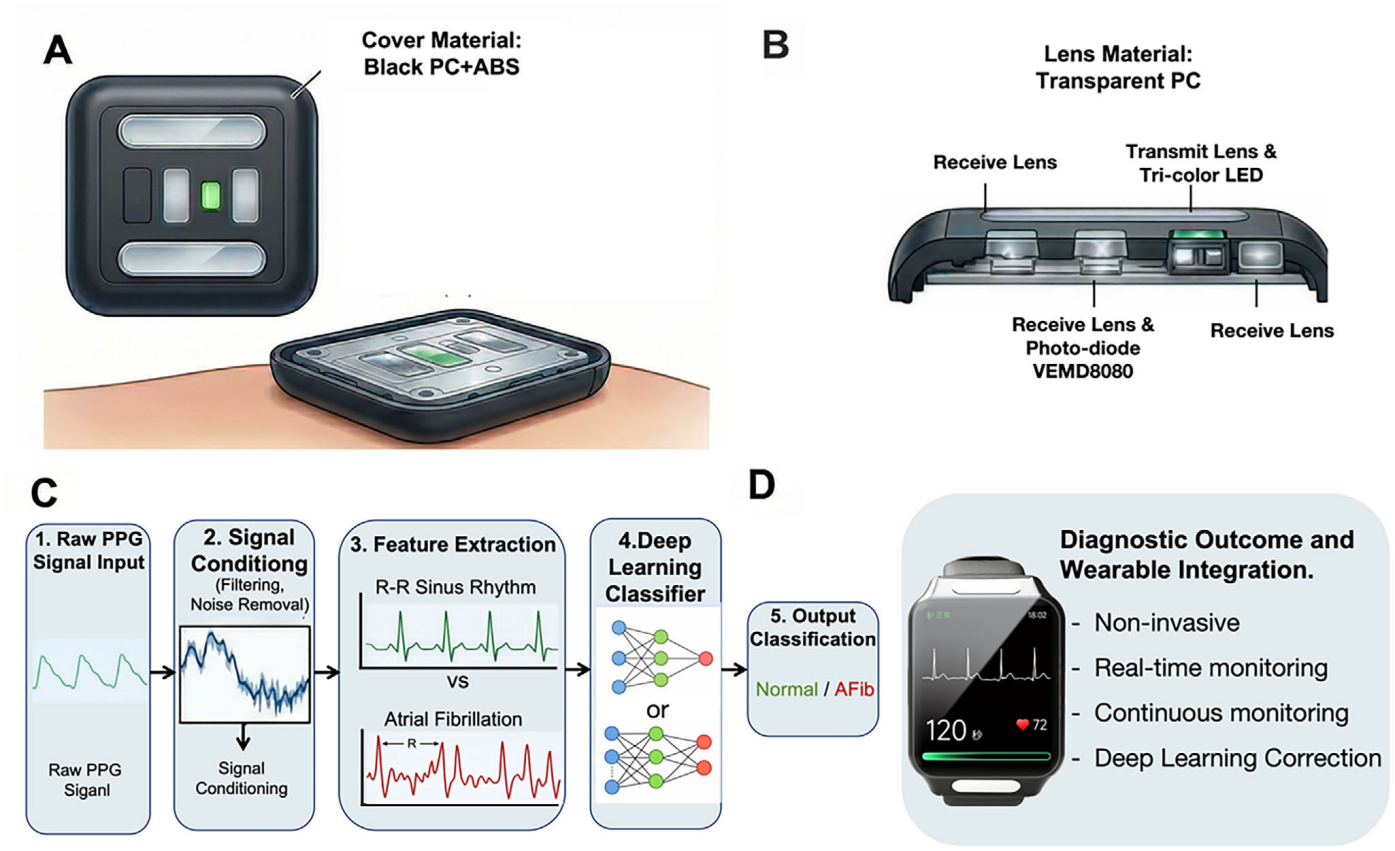
Smartwatch‐Based PPG Sensing and AI‐Based Atrial Fibrillation Detection Workflow. This figure illustrates the hardware configuration, optical signal acquisition, signal processing, and AI‐enabled AF classification of the smartwatch‐based PPG monitoring system. (A&B) Hardware Configuration. Top and side view of the smartwatch PPG sensor, showing the cover material (black PC+ABS), transparent PC lens, tri‐color LED transmitters, and photodiode detectors (VEMD8080) used for optical signal collection. (C) Signal Processing and AI Classification Workflow. Raw PPG signals undergo signal conditioning (filtering and noise removal), feature extraction (R‐R sinus rhythm vs atrial fibrillation), and deep learning classification to output AF or normal rhythm. (D) Diagnostic Outcome and Wearable Integration. Final classification output displayed on smartwatch interface, highlighting non‐invasive, real‐time, and continuous monitoring with AI‐based correction. Abbreviations: PPG: Photoplethysmography; AF: Atrial Fibrillation; PC: Polycarbonate; ABS: Acrylonitrile Butadiene Styrene.

By contrast, during AF, the rapid and disorganized atrial electrical activity leads to irregular conduction through the atrioventricular node, giving rise to an “absolutely irregular” ventricular response. This irregularity is directly reflected in the PPG signal as unpredictable fluctuations in pulse intervals and increased complexity of the temporal rhythm pattern. Meanwhile, owing to beat‐to‐beat variation in ventricular filling time and stroke volume, the pulse morphology often becomes unstable under AF. Such instability is manifested as irregular fluctuations in pulse amplitude, variation in the upslope and peak width, and inconsistency in the visibility and position of the dicrotic notch. In some cases, pulse deficit may also occur, where electrical depolarization is present on ECG, but peripheral perfusion is insufficient to generate a clear pulse wave, resulting in attenuated or even missing peaks in PPG.

Based on these features, AF identification from PPG generally relies on the combined recognition of interval irregularity and waveform inconsistency, with temporal alignment to W‐ECG used for window‐level verification to improve clinical interpretability and traceability. This multimodal collaborative strategy not only improves detection accuracy, but also reduces misclassification arising from the intrinsic limitations of a single signal modality.

#### 2.3.1 Core Sources of False‐Positive and False‐Negative Predictions

In wearable monitoring under free‐living conditions, errors in PPG‐based AF detection can be broadly attributed to two scenarios: irregular patterns not caused by AF, and insufficient or masked expression of AF‐related features. False‐positive predictions mainly arise from physical artifacts or physiological non‐AF arrhythmias. For example, arm motion, changes in skin–sensor contact pressure, or loose wearing may introduce motion artifacts, severe baseline drift, and peak jitter. In addition, frequent premature atrial contractions or premature ventricular contractions, bigeminy or trigeminy, and marked sinus arrhythmia, such as respiratory sinus arrhythmia, may produce genuine interval perturbation or rhythm irregularity. If classification is based primarily on interval irregularity without sufficient constraints from waveform morphology and signal quality, these conditions can be erroneously classified as AF (Figure 3C).

False‐negative predictions are more likely to occur when peripheral perfusion is poor, waveform amplitude is markedly reduced, or pulse deficit leads to an insufficient number of effective beats. Under such conditions, the irregularity of AF may be obscured by missed peak detection and waveform degradation, thereby preventing reliable recognition by the model. In addition, short paroxysmal AF episodes or transition states between AF and sinus rhythm may exhibit only atypical irregularity within a limited time window, making it difficult for the model to accumulate sufficient evidence for confident classification. Furthermore, temporal misalignment between PPG and ECG, inconsistency in annotation granularity across windows, and label noise may all compromise the effectiveness of supervised learning, thereby increasing the risk of missed detection and weakening model generalizability.

### 2.4 AI‐Enhanced Correction Model for PPG‐Assessed AF Burden

#### 2.4.1 Data Collection and Processing

##### 2.4.1.1 Multimodal Signal Acquisition

After participants wore the smartwatch and ECG monitoring device, the acquisition system continuously recorded two types of long‐term signals during daily activities, namely W‐PPG and P‐ECG. Meanwhile, short‐term W‐ECG signal acquisition was initiated through three triggering modes. First, symptom‐triggered acquisition was activated when participants experienced symptoms suggestive of arrhythmia, such as palpitations or chest discomfort. Second, scheduled acquisition was initiated according to predefined device strategies, including periodic scheduled recordings and event‐triggered recordings when real‐time W‐PPG analysis indicated a suspected positive episode. Third, user‐initiated measurement allowed participants to actively start W‐ECG recording through the smartwatch interface when they wished to perform an additional rhythm assessment. Once triggered, the system recorded W‐ECG signals. Because W‐ECG acquisition depends on factors such as participant compliance and electrode contact quality, it was regarded as intermittent data. To ensure that at least one complete rhythm assessment window was covered, each W‐ECG recording was required to last for no less than 30 s (Figure 4A).

**FIGURE 4:**
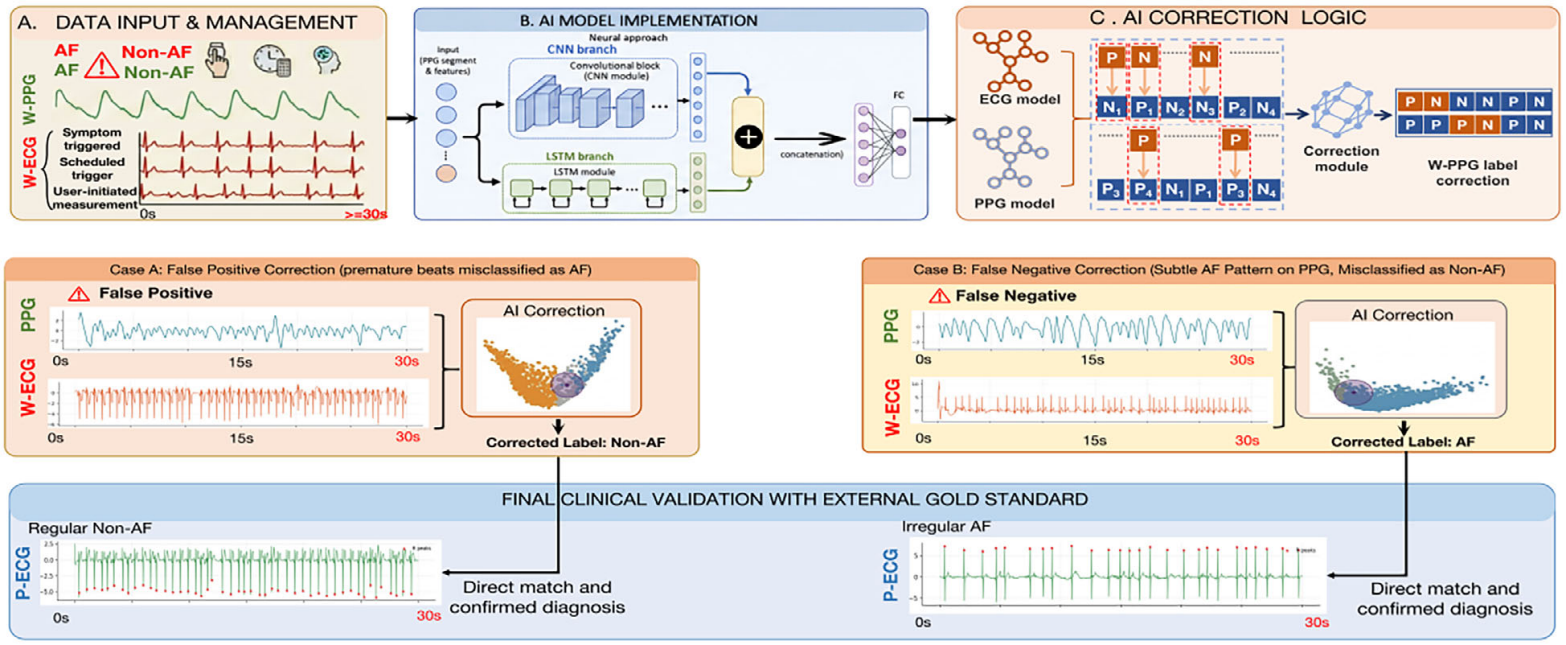
Workflow for AI‑enhanced AF burden estimation from wearable signals. This figure outlines the comprehensive process of data acquisition, model implementation, feature matching, and label correction to enhance the accuracy of AF burden assessment using wearable devices. (A) Data input and management. Continuous photoplethysmography (W‐PPG) and single‐lead electrocardiography (W‐ECG) are recorded by the smartwatch. User‐initiated triggers, algorithm‐detected PPG events and scheduled prompts ensure synchronous acquisition of W‐ECG segments for subsequent calibration. (B) AI model implementation. Continuous W‐PPG data are segmented and processed by a parallel CNN‐LSTM model. The CNN branch extracts local waveform morphology and short‐term structural features, whereas the LSTM branch captures long‐range temporal dependencies and rhythm dynamics. The feature representations from the two branches are concatenated and passed through a fully connected classification layer to output a predicted rhythm label, either atrial fibrillation (AF) or non‐AF, for each segment.(C) AI Correction logic. Feature vectors derived from the W‐PPG segments are compared with template features extracted from W‐ECG–synchronized epochs. Segments exhibiting high feature similarity to W‐ECG templates are identified, and the corresponding W‐PPG labels are corrected. W‐PPG labels are recalibrated by comparing the W‐PPG feature cluster to the W‐ECG label and the predefined P‐core (positive) and N‐core (negative) regions. Segments within the P‐core of a W‐ECG‐positive epoch are re‐labelled as positive (P), and segments within the N‐core of a W‐ECG‐negative epoch are re‐labelled as negative (N). Ambiguous segments (SPN sets) are resolved by specified criteria to minimize misclassification. Abbreviations: SENS: Sensitivity; SPEC: Specificity; AUC: Area Under the Curve; PPG: Photoplethysmography; ECG: Electrocardiogram; CNN: Convolutional Neural Networks and LSTM: Long Short‐Term Memory; P: positive; N: negative; SP: set P; SN: set N; SPN: set both.

##### 2.4.1.2 Signal Segmentation

For signal segmentation, W‐PPG and P‐ECG were divided chronologically into nonoverlapping 30 s segments. For continuously recorded W‐ECG signals obtained from a single acquisition event, a sliding‐window strategy with a step size of 1 s was adopted to generate multiple 30 s segments from the short continuous sequence, resulting in 29 s overlap between adjacent segments. This strategy was used to maximize data utilization and reduce the influence of occasional poor contact. Among the resulting segments, the one with the best signal quality was selected for subsequent correction analysis.

##### 2.4.1.3 Annotation of P‐ECG Segments

All 30‐s P‐ECG segments were independently annotated by two certified cardiologists blinded to PPG results and clinical data; disagreements were resolved by a third cardiologist. According to the reading results, each segment was assigned to one of three categories: AF, Non‐AF, or Invalid. Invalid segments generally resulted from signal noise, artifacts, or electrode detachment and could not be interpreted reliably; these segments were therefore excluded from subsequent training and evaluation.

##### 2.4.1.4 Quality Assessment and Filtering of W‐PPG Segments

To prevent low‐quality W‐PPG segments from introducing misleading rhythm features, signal quality assessment was performed before AF identification. Each 30 s W‐PPG segment was assigned a binary quality label, namely Qualified or Unqualified. First, segment‐wise Z‐score normalization was applied to reduce the influence of amplitude variation and baseline drift. Subsequently, a binary quality assessment model based on a one‐dimensional residual network was used to classify signal quality. The model took single‐channel sequences as input (shape: Batch Size, Channel = 1, Sequence Length = 768; sampling rate: 25.6 Hz) and extracted multiscale features through multiple groups of parallel one‐dimensional convolutional layers, ultimately outputting the predicted quality class. Based on the model output, W‐PPG segments predicted as unqualified were discarded, whereas the remaining qualified segments were retained for subsequent AF‐related feature extraction and matching analysis.

##### 2.4.1.5 Synchronous Segment Matching and Construction of Valid Samples

After W‐PPG quality control and manual annotation of P‐ECG were completed, W‐PPG and P‐ECG were synchronously matched based on 30 s time windows to construct paired segments. Only segment pairs satisfying all the following criteria were regarded as valid samples: the W‐PPG segment was qualified, the P‐ECG segment was interpretable rather than invalid, and the two signals were temporally aligned and complete. If any of these conditions was not met, for example, if the W‐PPG segment was unqualified, the P‐ECG segment was uninterpretable, temporal alignment failed, or data were incomplete, the corresponding segment pair was excluded from subsequent AF identification, feature matching, and correction procedures.

#### 2.4.2 Model Prediction

For W‐PPG segments that passed quality control (Qualified), segment‐level rhythm inference was performed using a deep learning model. The model generated a binary prediction for each segment, classifying it as either AF or Non‐AF (Figure 4B).

The model was built on a CNN–LSTM hybrid architecture (Figure 5), consisting of a CNN‐based morphological feature branch, an LSTM‐based temporal feature branch, and a fusion classification module. Both branches took the same W‐PPG segment as input but focused on complementary aspects of the signal. Specifically, the CNN branch was designed to capture local waveform morphology and short‐term structural patterns, whereas the LSTM branch was used to characterize rhythm dynamics and long‐range temporal dependencies across sequential time steps.

**FIGURE 5:**
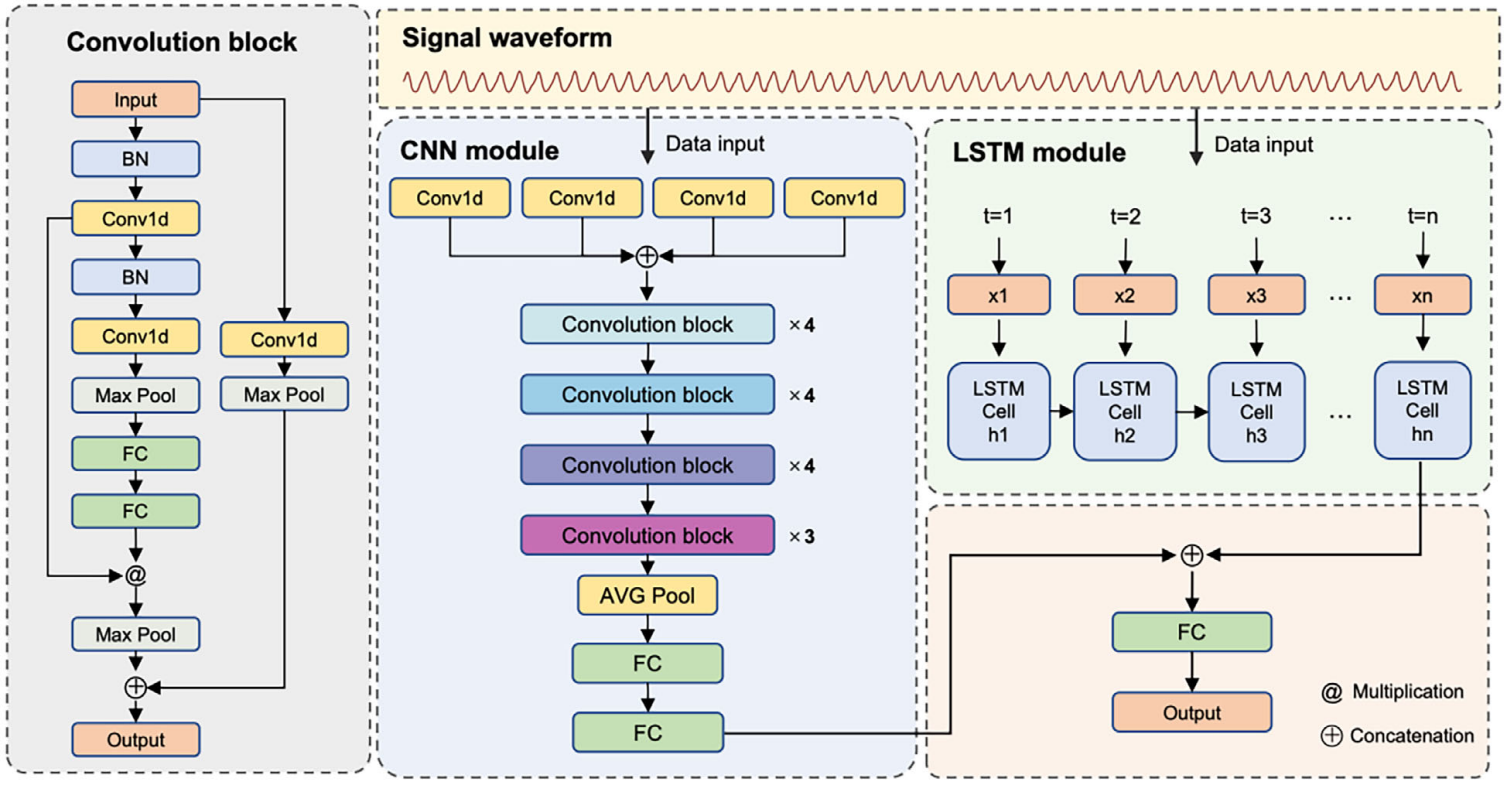
Architecture of the Convolutional–recurrent Neural Network used for AI‐assisted Correction of Smartwatch PPG signals in AF Burden Assessment. The raw PPG and single‐lead ECG waveforms are simultaneously fed into a deep learning framework composed of a CNN module and an LSTM module. The CNN module acts as a hierarchical feature extractor: four parallel 1D convolutional layers capture multi‐scale morphological patterns, followed by 15 convolution blocks for deeper representation learning. Each convolution block consists of BN, Conv1d, MaxPool, and residual connections. AVGPool, the features are passed through fully connected (FC) layers. The LSTM module is designed to capture the temporal dependencies within the sequential signals. At each time step, the input sequence is processed by LSTM cells, and the hidden states are propagated across time. The outputs of the CNN and LSTM branches are concatenated and further passed through FC layers to produce the final classification result. Abbreviations: PPG, Photoplethysmography; ECG, Electrocardiogram; CNN, Convolutional neural network; LSTM, long short‐term memory network; Conv1d, One‐dimensional convolution; BN, Batch normalization; MaxPool, Max pooling; AVGPool, Average pooling; FC, fully connected.

**FIGURE 6:**
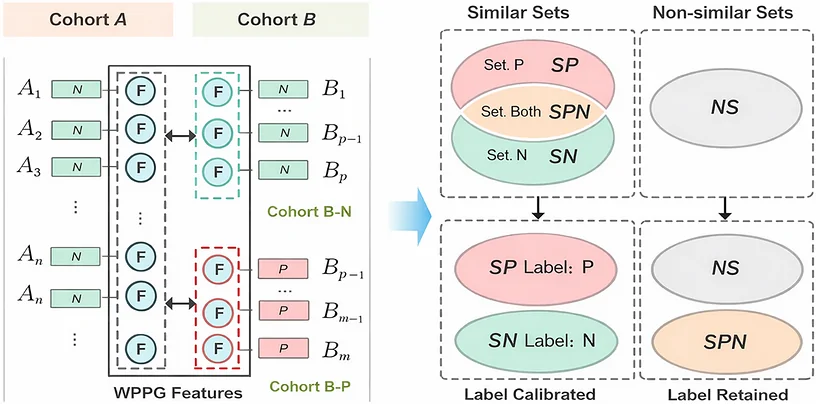
Feature Matching‐based Label Correction Framework for W‐PPG Segments. W‐PPG segments were divided into Cohort A and Cohort B according to the availability of concurrent W‐ECG recordings within the same acquisition window. Cohort A contained W‐PPG segments without paired W‐ECG and was used as the unlabeled set for calibration, whereas Cohort B contained synchronous W‐PPG segments with available W‐ECG and served as the template pool. Based on W‐ECG‐derived rhythm labels, Cohort B was further split into a positive template set (Cohort B‐P) and a negative template set (Cohort B‐N). For each segment in Cohort A, an intermediate feature representation, F (·), was extracted from the rhythm recognition model, and its similarity to the two template sets was quantified by nearest‐neighbor Euclidean distance. Using a predefined threshold (τ), samples were assigned to four subsets: SP, matched only to the positive template set; SN, matched only to the negative template set; SPN, matched to both template sets; and NS, matched to neither template set. Labels were then recalibrated according to the matching results: SP was reassigned as AF, SN as Non‐AF, whereas SPN and NS retained the original W‐PPG model‐predicted labels. Abbreviations: AF, atrial fibrillation; ECG, electrocardiogram; PPG, photoplethysmography; W‐ECG, wrist electrocardiogram; W‐PPG, wrist photoplethysmography; SP, samples matched only to the positive template set; SN, samples matched only to the negative template set; SPN, samples matched to both template sets; NS, non‐similar set; τ, similarity threshold.

The CNN branch employed four parallel 1D convolutional layers with different receptive fields to extract multiscale morphological features from the input signal. After channel‐wise fusion of the outputs from the parallel branches, the extracted features were fed into the convolutional backbone. The backbone consisted of multiple stacked convolution blocks. Each convolution block adopted a “BN–Conv1d–BN–Conv1d” unit structure and incorporated max‐pooling for down sampling. In addition, a residual shortcut pathway was introduced, in which the input was transformed through a matching operation, including Conv1d and MaxPool when necessary, and then added to the output of the main branch. This design ensured dimensional consistency while facilitating effective information and gradient propagation. The backbone output was subsequently aggregated along the temporal dimension by global average pooling to generate a fixed‐length segment‐level vector, which was further transformed by two fully connected layers to obtain the CNN‐derived representation of overall morphological patterns.

To model temporal dependencies, an LSTM module was introduced to characterize rhythm evolution and long‐range sequential patterns within each segment. The raw sequence was recursively processed over time steps to update hidden states, thereby encoding temporal information such as beat‐to‐beat interval fluctuation, persistence of rhythm irregularity, and variation trends across the segment. In this way, the LSTM branch complemented the CNN branch by focusing not on local morphology, but on sequential rhythm dynamics. The LSTM branch ultimately generated a segment‐level temporal representation for subsequent fusion with the CNN‐derived morphological representation.

During feature fusion, the feature representations from the CNN and LSTM branches were concatenated to integrate morphological and temporal information into a fused representation vector. This fused vector was fed into the fully connected classification head, which output the probability that the segment belonged to AF or Non‐AF, together with the corresponding predicted class. In this manner, an independent prediction was obtained for each W‐PPG segment.

For W‐ECG signals, segment‐level rhythm inference was performed using the same CNN–LSTM architecture. The model structure, feature fusion strategy, and classification head were kept consistent with those used for W‐PPG, and the model output was likewise a binary classification result, namely AF or Non‐AF.

Among the 1,054 patients included in the analysis, 2,165,338 ECG‐labeled W‐PPG segments were obtained. Of these, 65,318 segments were excluded due to incomplete device wearing, leaving 2,100,020 evaluable W‐PPG segments. After signal quality control, 1,296,932 segments were classified as qualified and included in the final analysis, whereas 803,088 segments were identified as low‐quality W‐PPG segments and excluded. Accordingly, the proportion of low‐quality W‐PPG segments was 38.2%, and the final valid inclusion proportion was 61.8%. Among the 1,296,932 qualified W‐PPG segments, W‐ECG–based correction changed the original W‐PPG label in 4,985 segments.

The corrected segments included two types of label changes. First, 1,849 W‐PPG segments were corrected from Non‐AF to AF. Compared with the ECG reference standard, 1,588 of these segments were classified as false negatives by W‐PPG before correction and were subsequently corrected to true positives. Second, 3,136 W‐PPG segments were corrected from AF to Non‐AF. Compared with the ECG reference standard, 2,686 of these segments were classified as false positives by W‐PPG before correction and were subsequently corrected to true negatives.

It should be noted that the CNN–LSTM architecture itself is a well‐established time‐series modeling framework and was not intended to represent the primary novelty of this study. In the present work, CNN modules were used to extract local morphological features from wearable ECG and PPG segments, whereas LSTM units were used to capture temporal dependencies across sequential signal patterns. The main innovation lies in the dual‐modal AF burden assessment strategy, in which continuous W‐PPG monitoring provides long‐duration rhythm surveillance and intermittent W‐ECG recordings serve as high‐fidelity reference anchors for correcting PPG‐derived AF burden estimation. This ECG‐anchored correction framework aims to improve quantitative AF burden assessment by reducing PPG misclassification caused by noise, premature beats, and other irregular non‐AF rhythms.

In the current implementation, W‐PPG and W‐ECG signals were acquired in real time by the smartwatch, while segment‐level prediction and ECG‐anchored correction were performed on the cloud platform. Therefore, the system was not designed to provide instant real‐time AF burden output. Instead, its intended clinical workflow was to generate the previous day's AF burden result before the daily clinical assessment. This design is consistent with the clinical objective of longitudinal AF burden quantification rather than immediate arrhythmia alarm generation.

#### 2.4.3 Feature Matching and Label Correction (Figures 4C and 6)

##### 2.4.3.1 Sample Grouping

According to whether W‐ECG was available within the same acquisition window, W‐PPG segments were divided into two cohorts. The first was Cohort A (to‐be‐corrected set), which comprised W‐PPG segments acquired in time windows without corresponding W‐ECG recordings. The second was Cohort B (template set), which consisted of synchronized W‐PPG segments collected in time windows where W‐ECG was simultaneously recorded and was therefore used as the source of template samples for feature matching.

##### 2.4.3.2 Template Construction and Class Partitioning

For template construction, the predicted labels of W‐ECG were used as the reference to further divide the synchronized W‐PPG segments in Cohort B into two subsets. Specifically, Cohort B‐P (positive template set) comprised W‐PPG segments whose corresponding W‐ECG segments were predicted as AF, whereas Cohort B‐N (negative template set) comprised W‐PPG segments whose corresponding W‐ECG segments were predicted as Non‐AF.

##### 2.4.3.3 Feature Extraction and Similarity Measurement

For each W‐PPG segment, an intermediate segment‐level representation vector, denoted as F(·), was extracted from the rhythm recognition model. For any sample x∈Cohort A, its similarity to the two template subsets was quantified using Euclidean distance:

(1) $$d_{P}\;\left(x\right)=\min_{y\in B-P}|F\left(x\right)-F\left(y\right)\;{|}_{2},$$

(2) $$d_{N}\;\left(x\right)=\min_{y\in B-N}|F\left(x\right)-F\left(y\right)\;{|}_{2}$$

Here, the min operator denotes the nearest‐neighbor distance, that is, the minimum distance from sample x to a given template set. A matching threshold τ was then introduced, and a segment was considered to be matched to a template set when d(·)< τ.

Based on these decision rules, W‐PPG segments in Cohort A were further divided into four subsets:

- Set P(SP): (3)*d_P_* (*x*) < τ and *d_N_* (*x*) ≥ τ
- Set N(SN): (4)*d_N_* (*x*) < τ and *d_P_* (*x*) ≥ τ
- Set Both(SPN):(5)*d_N_* (*x*) < τ and *d_P_* (*x*) < τ
- Set NS(NS): (6)*d_N_* (*x*) ≥ τ and *d_P_* (*x*) ≥ τ

##### 2.4.3.4 Correction Decision

According to the above subset assignment, label correction was performed using the following rules. For Set P (SP), the segment label was corrected to AF. For Set N (SN), the segment label was corrected to Non‐AF. For Set Both (SPN) and Set NS (NS), no correction was applied, and the original prediction generated by the W‐PPG model was retained.

For visualization of the similarity relationships, the high‐dimensional W‐PPG features used for matching were projected into a two‐dimensional space to generate a schematic 2D representation, in which the x‐ and y‐axes correspond to different feature dimensions (Figure 7).

**FIGURE 7:**
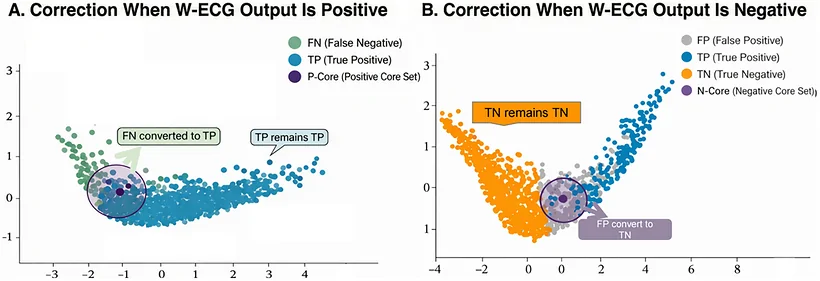
Correction Effects of W‐ECG–W‐PPG Template Similarity Matching. (A) When the W‐ECG classifier outputs a positive result, the circle centered at the P‐core denotes the region of W‐PPG templates that, according to similarity matching, can be re‐labeled as positive. The samples within this circle consist of false negatives (FN; green) and true positives (TP; blue). After calibration, FN are converted to TP, eliminating missed detections, while TP remain positive, leaving correct predictions unchanged. Overall, this procedure converts FN into TP and enhances classification accuracy. (B) When the W‐ECG classifier outputs a negative result, the circle centered at the N‐core represents the range of W‐PPG templates that can be corrected to the negative label. Samples inside this region include false positives (FP; grey), true positives (TP; blue) and true negatives (TN; orange). Following calibration, TN retain their label, FP are re‐classified as TN, and TP are re‐classified as false negatives (FN). Because the proportion of FP in this region is markedly larger than that of TN, the ratio of correct adjustments (FP→TN) exceeds that of incorrect adjustments (TP→FN), and the calibration still yields a net improvement. Abbreviations: W‐ECG: Watch‐based Electrocardiogram; PPG: Photoplethysmography; AF: Atrial Fibrillation; TP: True Positive; TN: True Negative; FP: False Positive; P‐Core: Positive Core Set; N‐Core: Negative Core Set.

To comprehensively evaluate the performance of the AI‐enhanced correction framework, multiple metrics were adopted, including the area under the receiver operating characteristic curve (AUC), sensitivity (SE), specificity (SP), positive predictive value (PPV), negative predictive value (NPV), accuracy (ACC), and F1 score. As summarized in the radar plot (Figure 1D), the model showed consistently strong performance across all evaluation metrics. Specifically, after dual‐modal correction, the AUC reached 0.9898, with an SE of 0.9860 and an SP of 0.9927. The PPV and NPV were 0.9896 and 0.9902, respectively, while the overall ACC reached 0.9899 and the F1 score reached 0.9878. These findings indicate that the proposed correction framework achieves high sensitivity, specificity, and overall stability for AF burden assessment in practical smartwatch‐based monitoring.

It should be emphasized, however, that the small numerical differences between the metrics before and after AI correction in the individual‐level radar plot should be interpreted with caution. The high agreement between the wearable system and the patch ECG reference does not imply that PPG signals are intrinsically equivalent to ECG or are unaffected by motion artifacts. Rather, the radar plot summarizes average performance across the entire validation cohort, in which segments with severe PPG misclassification accounted for only a limited proportion. The main benefit of ECG‐anchored correction is expected to occur in error‐prone patients or signal segments, particularly those affected by premature atrial or ventricular beats, irregular non‐AF rhythms, or reduced PPG signal quality. Because these cases represent only a subset of the overall cohort, their correction may be diluted in aggregate performance metrics and therefore appear numerically modest at the individual level. Thus, the present results should be interpreted as demonstrating high individual‐level agreement with patch ECG for AF burden assessment, rather than complete equivalence between PPG‐based monitoring and ECG. Further subgroup‐focused analyses are warranted to evaluate the specific benefit of AI correction in patients with frequent ectopic beats, irregular non‐AF rhythms, or poorer PPG signal quality.

### 2.5 Clinical Application of the Dual‐Modal Wearable AF Monitoring System

The AI‐enhanced dual‐modal wearable monitoring system developed in this study, which integrates a CNN–LSTM framework with multimodal physiological signals including PPG and single‐lead ECG, markedly improved the accuracy and reliability of AF burden assessment. This strategy provides a technical foundation for shifting clinical AF management from conventional event identification toward quantitative and dynamic burden‐guided management. First, in the context of early screening for high‐risk populations, the system enables effective detection of clinically prevalent yet often occult AF through high‐accuracy real‐time correction. Because AF is highly intermittent in nature, conventional 12‐lead ECG or short‐term Holter monitoring often carries a substantial risk of missed diagnosis. By achieving a sensitivity of 98.60% and a specificity of 99.27%, the proposed system enables high‐fidelity capture of arrhythmic events during daily activities, thereby creating a valuable clinical window for earlier intervention in populations at elevated risk of stroke.

In nonsurgical patients, long‐term AF burden monitoring is essential for tracking disease progression and guiding therapy. Unlike traditional binary (present/absent) assessment, this study quantifies the cumulative proportion of time in AF. This long‐term monitoring capability allows clinicians to track the temporal trajectory of AF burden in real time and thereby achieve more refined disease assessment in nonsurgical settings. This is particularly relevant to anticoagulation management, where the clinical challenge often lies in balancing thromboembolic prevention against bleeding risk. By leveraging AF burden data generated by this system, a more individualized assessment may become possible. For patients with persistently high AF burden, initiation or intensification of anticoagulation may be more strongly justified to reduce stroke risk, whereas for those with extremely low burden or prolonged absence of detectable AF, anticoagulation intensity may potentially be adjusted under close surveillance, thereby minimizing bleeding complications while maintaining protection against thromboembolic events.

The system also provides a reliable basis for evaluating the effectiveness of clinical interventions, particularly after AF ablation, and may support subsequent anticoagulation decision‐making. In this context, procedural success is no longer judged solely based on short‐term postprocedural ECG follow‐up but can instead be assessed through long‐term dual‐modal monitoring to determine whether AF recurrence occurs and, if so, to what extent recurrence contributes to the overall AF burden. If high‐accuracy monitoring confirms that post‐ablation AF burden remains persistently at a very low level, this may provide stronger evidence to support clinical decisions regarding the safety of anticoagulant discontinuation. Notably, AI‐based correction reduced the mean absolute percentage error from 1.11% to 0.85%, corresponding to a 23.4% reduction (Figure 8). This substantial gain in quantitative precision may further enable the development of future embolic‐risk assessment frameworks grounded in AF burden rather than conventional categorical rhythm assessment alone.

**FIGURE 8:**
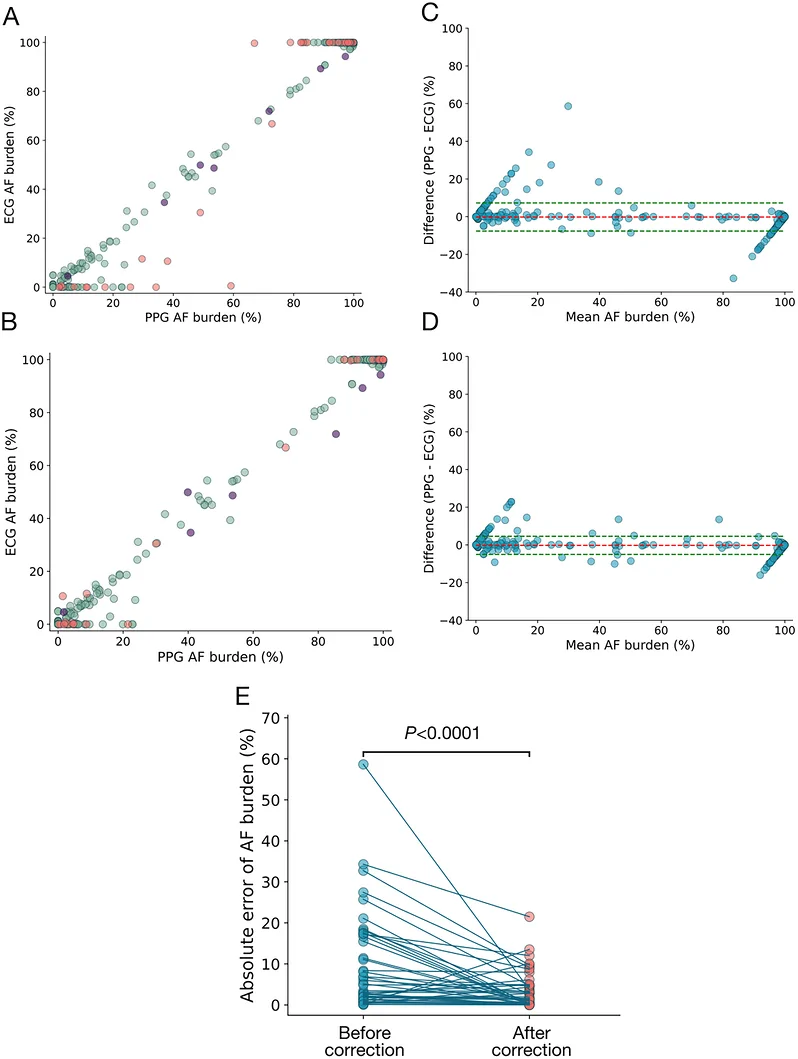
Scatter and Bland‐Altman Plots for W‐PPG Correction by W‐ECG. Comparison of AF burden estimated from wearable PPG recordings and reference ECG measurements. (A) Correlation analysis before correction. (B) Correlation analysis after correction. In panels A and B, the x‐axis represents AF burden estimated by the wearable device (PPG AF burden, %) and the y‐axis represents AF burden measured by the reference ECG (ECG AF burden, %). (C) Bland–Altman analysis before correction. (D) Bland–Altman analysis after correction. In panels C and D, the x‐axis represents the mean AF burden (%) obtained from the wearable device and the reference ECG, and the y‐axis represents the difference in AF burden (%) between the wearable estimate and the reference ECG (PPG − ECG). (E) Comparison of absolute AF burden error before and after correction. All 1,054 participants were included in the analyses. Because multiple participants had similar or identical AF burden values, some data points overlap in the plots, resulting in fewer visually distinguishable points than the actual sample size. The figure was generated using enhanced visualization settings to improve the display of overlapping observations. Abbreviations: PPG: Photoplethysmography; ECG: Electrocardiogram.

In the longer term, AF burden may emerge as an important complement to, or even a major refinement of, the current CHA2DS2‐VASc‐based paradigm [50, 51]. Existing clinical scoring systems are primarily based on static risk factors, such as age and hypertension history, but do not account for dynamic differences in AF activity. For example, two patients with identical hypertension history and the same age of 70 years would each receive a CHA2DS2‐VASc score of 2, and current guideline‐based strategies would generally recommend a similar anticoagulation approach. However, if monitoring with the present system reveals that one patient has an AF burden of only 1%, whereas the other has a burden approaching 100%, their actual thromboembolic risk profiles are unlikely to be equivalent. By quantitatively capturing this previously concealed risk heterogeneity, the present technological framework points toward a more precise, accessible, and individualized era of AF management, and may provide a key enabling tool for the future transformation of stroke prevention strategies.

However, AF burden mainly reflects the total amount of AF and does not fully capture the temporal distribution of AF episodes. Patients with similar AF burden may have different recurrence patterns, such as a single clustered episode or multiple intermittent episodes. Therefore, newer biomarkers, including AF Density [52] and AF Aggregation [53], have been proposed to describe the temporal organization and clustering of AF episodes. AF Density characterizes the degree of temporal aggregation of AF burden, whereas AF Aggregation evaluates the deviation of the observed AF episode distribution from a uniformly distributed pattern. These metrics may provide complementary information beyond conventional AF burden. Although AF Density and AF Aggregation were not directly calculated in the present study, the corrected continuous segment‐level rhythm outputs generated by our framework may provide a basis for deriving these temporal‐pattern biomarkers in future studies.

## 3 Conclusions

In conclusion, this study demonstrates that an AI‐enhanced dual‐modal wearable framework integrating continuous W‐PPG monitoring with intermittent single‐lead W‐ECG correction can improve the accuracy and reliability of AF burden assessment. By using W‐ECG as a high‐confidence reference to correct W‐PPG‐derived rhythm predictions, the proposed system reduced false‐positive and false‐negative classifications, achieved high diagnostic performance, and decreased the mean absolute percentage error of AF burden estimation by 23.4%. These findings support the potential of multimodal wearable monitoring for scalable, noninvasive, and quantitative AF management. Although further multicenter validation in broader populations is warranted, this framework provides a practical technical foundation for continuous AF burden monitoring and future burden‐guided personalized care.

Despite these promising results, several limitations should be acknowledged. First, this study was conducted at a single center in patients undergoing AF ablation, predominantly comprising individuals with paroxysmal or persistent AF and an overall low AF burden, which may limit its generalizability to newly diagnosed AF or long‐standing persistent AF populations. Second, the ECG‐based correction mechanism relied on patient‐initiated recordings or algorithm‐prompted W‐ECG acquisition and may therefore be susceptible to compliance‐related bias, as adherence can vary substantially in real‐world settings. Third, although patient‐level cross‐validation was performed, multicenter external validation is warranted to confirm the robustness of the proposed framework across diverse ethnic backgrounds, comorbidity profiles, and device platforms. Fourth, the label‐correction strategy, while improving classification consistency by leveraging W‐ECG‐derived template information, cannot fully resolve all ambiguous segments. In particular, for segments categorized as SetBoth or SetNS, in which features either match multiple templates or fail to match any template, the system reverts to the original PPG‐based prediction when reliable correction is not feasible. Given the susceptibility of PPG signals to noise and motion artifacts, these fallback cases may still introduce misclassifications.

## 4 Experimental Section

### 4.1 Device Specifications and Standardized Wearing Protocol

The hardware system used in this study consisted of two components: a single‐lead patch‐based ECG recorder (P‐ECG) and a wrist‐worn smart sensing terminal integrating W‐ECG and W‐PPG. All devices were provided by Chengdu Xinjikang Technology Co., Ltd. To ensure data accuracy and minimize motion‐induced artifacts in W‐PPG acquisition, all devices were fitted with the assistance of trained clinical research staff.

For P‐ECG placement, after standardized skin preparation, the flexible electrode patch was positioned precisely over the left precordial region, corresponding to the V2–V3 lead area. Skin preparation included hair removal and degreasing with 75% alcohol to minimize signal interference during acquisition. This procedure ensured stable operation of the P‐ECG device at a sampling frequency of 250 Hz, thereby supporting high‐quality ECG recording.

For smartwatch placement, the device was worn on the nondominant wrist and positioned away from the ulnar styloid process to improve wearing comfort. During device fitting, W‐PPG was continuously sampled at 25.6 Hz, whereas W‐ECG was recorded on demand at 512 Hz through dry stainless‐steel electrodes located on the back and side of the watch case. A closed circuit was formed when the participant touched the side electrode with the opposite hand, enabling single‐lead W‐ECG acquisition. Furthermore, patients were equipped with the dual‐modal wearable device within 24 hours before the catheter ablation procedure, and monitoring was continued until hospital discharge. The devices were temporarily removed during the ablation procedure and reapplied after the procedure when clinically appropriate. The mean total ECG acquisition duration was 27.03 ± 3.67 hours (range: 8.30–46.48 hours), and the mean total PPG acquisition duration was 22.60 ± 4.82 hours (range: 6.24–42.12 hours).

### 4.2 Data Segmentation and Automated Algorithmic Analysis

The P‐ECG and W‐PPG data streams were segmented into nonoverlapping 30 s windows. To further improve signal quality and maximize the utility of short‐duration recordings, W‐ECG segments were generated using a sliding‐window strategy with 29 s overlap and were jointly analyzed with the corresponding W‐PPG segments. During this process, the segment with the best signal quality and valid temporal correspondence was selected for integrated diagnosis.

All 30 s segments were automatically classified using a predefined AF detection algorithm. W‐ECG outputs were categorized as AF or non‐AF. AF burden was calculated as the proportion of AF segments among all valid monitoring segments. To evaluate the accuracy of the automated algorithm, all 30‐s P‐ECG segment were independently reviewed in a blinded manner by two senior cardiologists, and disagreements were adjudicated by a third expert when necessary.

### 4.3 Statistical Analysis

All statistical analyses were performed using IBM SPSS Statistics 29.0. Continuous variables were expressed as mean ± standard deviation (SD) or standard error (SE), as appropriate according to data distribution, whereas categorical variables were presented as frequencies and percentages. Sensitivity, specificity, and AUC of W‐PPG before and after correction were evaluated using 2 × 2 contingency tables in combination with generalized linear regression models, with within‐subject clustering incorporated to account for interindividual variation. Pearson correlation analysis was used to assess heart‐rate consistency, and Bland–Altman analysis was performed to evaluate bias and the 95% limits of agreement between the two monitoring systems. A two‐sided P value < 0.05 was considered statistically significant.

### 4.4 Large‐Scale Clinical Validation Cohort

To validate device performance in a real‐world setting, patients undergoing AF ablation at Beijing Anzhen Hospital between January and June 2024 were recruited. Inclusion criteria were age ≥18 years and provision of written informed consent. Exclusion criteria included implanted pacemakers and allergy to wearable‐device materials. A total of 1165 participants were initially recruited, and 1054 patients were ultimately included in the final analysis (mean age, 62.1 years; 61.2% male). Among them, 579 patients (54.9%) had paroxysmal AF and 461 (43.7%) had persistent AF. Comorbidities included hypertension in 50.4% and diabetes mellitus in 19.5% of patients. Detailed baseline characteristics were collected from the electronic medical record (EMR) system and were summarized in Table 1. The study protocol was approved by the Ethics Committee of Beijing Anzhen Hospital (approval No. KS2022030). All participants provided written informed consent before enrollment.

**TABLE 1:** Baseline Characteristics.

Baseline Characteristics	N = 1054
Age (years)	62.1 ±10.4
Sex (male)	645 (61.2%)
BMI (kg/m^2^)	26.5 ±3.5
AF type	
Paroxysmal AF	579 (54.9%)
Persistent AF	461 (43.7%)
Others	14 (1.3%)
Hypertension	531 (50.4%)
Stroke	96 (9.1%)
Diabetes	206 (19.5%)
Congestive heart failure	131 (12.4%)
Arterial vascular disease	42 (4.0%)
Coronary artery disease	151 (14.3%)
Hyperlipidemia	331 (31.4%)
Repeat ablation	134 (12.7%)
SBP	129.8 ±16.1
DBP	79.9 ±10.3
PPG‐measured burden	9.7% (IQR: 0%‐99.5%)

Data are presented as mean ± SD, n (%), or median (IQR). Abbreviations: BMI: Body Mass Index; SBP: Systolic Blood Pressure; DBP: Diastolic Blood Pressure; PPG: Photoplethysmography; ECG: Electrocardiogram

### 4.5 Stringent Quality Control and Signal Stability Assessment

To ensure high data quality, strict exclusion criteria were applied for signal quality control. In total, 111 participants were excluded, including 7 for missing data, 9 for effective wearing time <2 h, and 95 for poor signal quality. Poor ECG quality was defined as baseline drift resulting in loss of identifiable QRS complexes. Poor PPG quality was defined as severe waveform distortion caused by vigorous motion, such as arm movement, or improper wearing, including an excessively loose or overly tight fit. Notably, signal stability was significantly better during nighttime than during daytime activity, with a higher proportion of valid recordings, thereby providing a more reliable basis for 24 h AF burden assessment.

### 4.6 Ethics and Compliance Statement

Data collection was conducted in strict accordance with the Declaration of Helsinki and its subsequent amendments. The study protocol was approved by the Ethics Committee of Beijing Anzhen Hospital. All participants provided written informed consent before enrollment after being fully informed of the study objectives, monitoring procedures, potential risks, and the anonymization strategy applied to their data.

## Author Contributions

**S.Z**., **J.L.W**., **W.Z**., and **C.M**. conceptualized the study and designed the experiments. **S.Z**., **J.L.W**., **X.W**., and **H.F**. contributed to the study methodology and implementation. **S.Z**., **Y.Z**., **F.L**., **S.W**., **L.Z**., **X.K**., **J.W**., **L.H**., **S.X**., **X.L**., **J.Z**., **X.L**., **X.G**., **N.Z**., **S.L**., **C.J**., **R.T**., **C.S**., **D.L**., **X.D**., and **J.D**. contributed to patient enrollment, clinical investigation, and data collection. **S.Z**., **J.L.W**., **X.W**., and **W.Z**. conducted the data analysis and interpretation. **S.Z**. and **J.L.W**. completed the manuscript writing, with guidance from **X.W**., **H.F**., **W.Z**., and **C.M. W.Z**. and **C.M**. supervised the overall study. All authors reviewed and approved the final manuscript.

## Conflicts of Interest

The authors declare no conflicts of interest.

## Data Availability

The data that support the findings of this study are available from the corresponding author upon reasonable request.
